# Activin B Promotes Epithelial Wound Healing In Vivo through RhoA-JNK Signaling Pathway

**DOI:** 10.1371/journal.pone.0025143

**Published:** 2011-09-19

**Authors:** Min Zhang, Nu-Yun Liu, Xue-Er Wang, Ying-Hua Chen, Qing-Lin Li, Kang-Rong Lu, Li Sun, Qin Jia, Lu Zhang, Lin Zhang

**Affiliations:** 1 Department of Histology and Embryology, Southern Medical University, Guangzhou, China; 2 Key Laboratory of Functional Proteomics of Guangdong Province, Department of Pathophysiology, Southern Medical University, Guangzhou, China; University of Hong Kong, Hong Kong

## Abstract

**Background:**

Activin B has been reported to promote the proliferation and migration of keratinocytes in vitro via the RhoA-JNK signaling pathway, whereas its in vivo role and mechanism in wound healing process has not yet been elucidated.

**Principal Findings:**

In this study, we explored the potential mechanism by which activin B induces epithelial wound healing in mice. Recombinant lentiviral plasmids, with RhoA (N19) and RhoA (L63) were used to infect wounded KM mice. The wound healing process was monitored after different treatments. Activin B-induced cell proliferation on the wounded skin was visualized by electron microscopy and analyzed by 5′-bromodeoxyuridine (BrdU) incorporation assay. Protein expression of p-JNK or p-cJun was determined by immunohistochemical staining and immunoblotting analysis. Activin B efficiently stimulated the proliferation of keratinocytes and hair follicle cells at the wound area and promoted wound closure. RhoA positively regulated activin B-induced wound healing by up-regulating the expression of p-JNK and p-cJun. Moreover, suppression of RhoA activation delayed activin B-induced wound healing, while JNK inhibition recapitulated phenotypes of RhoA inhibition on wound healing.

**Conclusion:**

These results demonstrate that activin B promotes epithelial wound closure in vivo through the RhoA-Rock-JNK-cJun signaling pathway, providing novel insight into the essential role of activin B in the therapy of wound repair.

## Introduction

Cutaneous wound repair is a complex process, which involves a series of biological events involving inflammation, new tissue formation and tissue remodeling [1]. During the process of new tissue formation, the proliferation and migration of keratinocytes is required for wound re-epithelialization and healing [2]. Various growth factors and cytokines have been found to play a critical role in wound healing [3]. Among these factors, activins, which are members of the transforming growth factor β (TGF-β) superfamily, play an important role in regulating normal function of epithelial cells, skin development and wound healing [4], [5]. Three different forms of activin have been identified, including the homodimers activin A (βAβA), activin B (βBβB) and the heterodimer activin AB (βAβB) [6]. Strong and persistent induction of activin B have been found in the hyperproliferative epithelia at the wound edge and in the migrating epithelia of the tongue [4]. In addition, overexpression of the activin antagonist follistatin or a dominant-negative activin receptor IB mutant (dnActRIB) delayed wound re-epithelialization after skin injury in mice [7], [8], implying a critical role of activin B in the regulation of wound healing.

Although the significance of activin B in wound closure has been recognized, the mechanism by which activin B mediates wound re-epithelialization and promotes wound healing after injury remains poorly understood. Accumulating *in vitro* and *in vivo* evidence has shown that mitogen-activated protein kinase kinase (MEK) kinase 1 (MEKK1)-driven c-Jun N-terminal kinase (JNK) activation contributes to activin-stimulated actin stress fiber formation, c-Jun phosphorylation and cell migration [9], [10], [11]. Furthermore, activin-stimulated activation of MEKK1-JNK-cJun signal pathway depends on the small G protein RhoA in the regulation of actin stress fiber formation, actin cytoskeleton reorganization and subsequent *in vitro* keratinocyte migration [9]. Nevertheless, the *in vivo* role of activin B and the molecular mechanism by which activin B regulates wound repair are still unclear.

In the present study, we investigated the effect of RhoA activation on activin B-stimulated wound closure in KM mice by using infection of the cells with dominant negative or constitutively active RhoA expression constructs. The results showed that RhoA positively mediated activin B-induced wound healing by induction of JNK and cJun expression. Moreover, SP600125, a specific JNK inhibitor, efficiently suppressed RhoA activation-mediated wound healing through an activin B mediated process, and the activin antagonist-follistatin remarkably blocked activin B-induced wound healing.

## Results

### Activin B promotes wound closure

**Figure 1:**
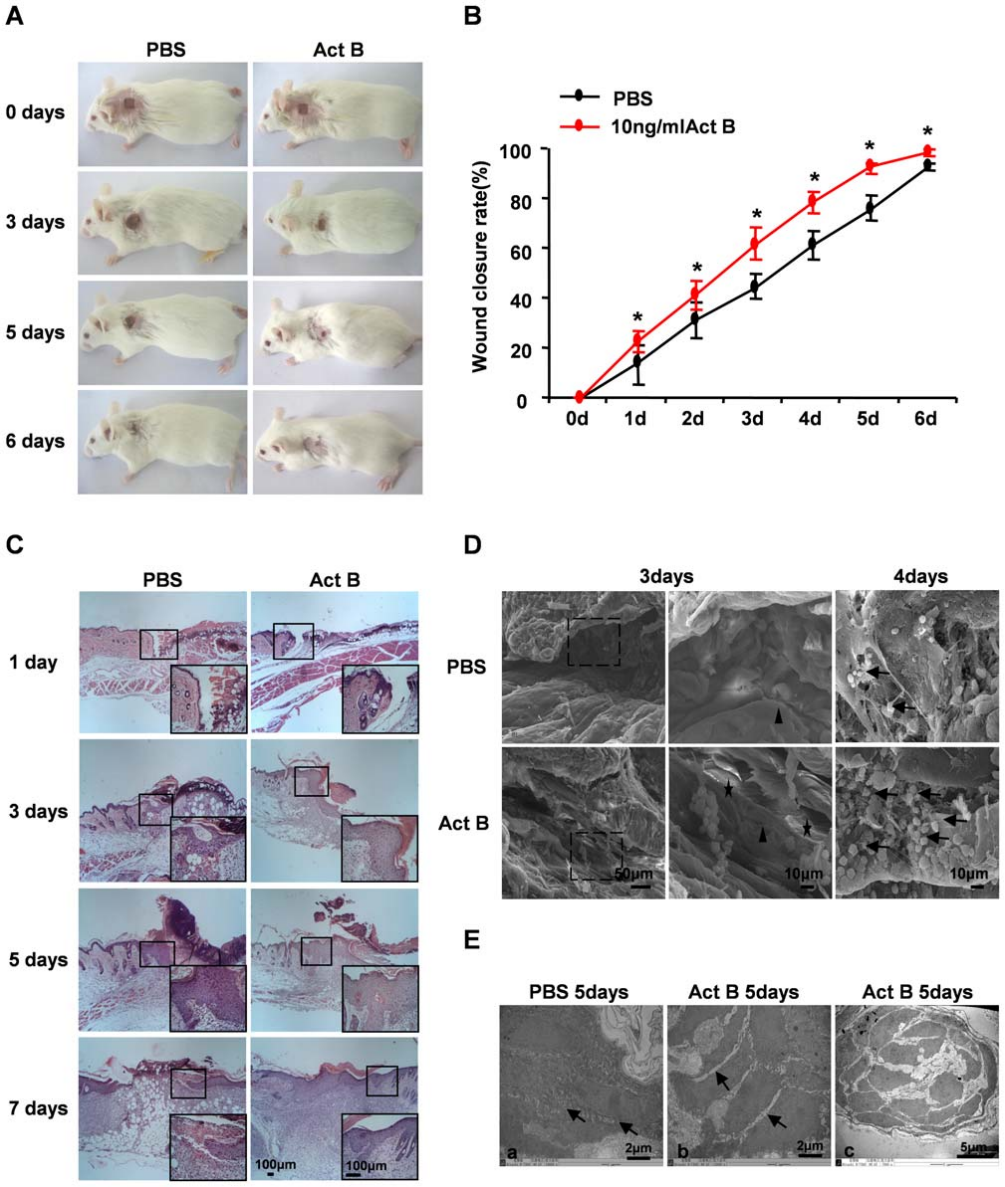
Time course of wound closure promoted by Activin B. Forty four mice were randomly assigned to PBS group and Act B group (n = 22). (A) Activin B promoted wound closure in KM mice as compared with control. KM mice received 0.2 ml of PBS or Activin B treatment three times per day. The wound healing status was monitored every day. (B) The wound closure rate was calculated as described in Materials and Methods. *P<0.01compared with PBS control. (C) H&E staining of the skin tissues surrounding wound sites. Scale bar: 100 µm. (D) SEM analysis showed that activin B induced cell proliferation on the surface (black star) and cross-section (black arrows) of wound area. Re-epithelized squamous epithelium is indicated by black arrowheads. (E) TEM analysis demonstrated that Activin B reduced cell-cell contacts and increased intercellular spaces (arrows) at wound area (b) compared with control (a). (c) The hair follicle seen in the regenerated skin after activin B treatment. Ea, Eb, Scale bar: 2 µm; Ec, Scale bar: 5 µm.

After wound creation, mice were randomly assigned to two groups; control and activin B group. Control group mice received a 0.2 ml of PBS (pH 7.4) applied to the site surrounding the wound three times a day. To induce wound healing, the same volume of activin B (10 ng/ml) was administered to the wounded skin. Compared with control, activin B promoted wound closure (Fig. 1A). One day following treatment wound closure was significantly higher with activin B treatment, and reached over 99% after 6 days (Fig. 1B). Hematoxylin and eosin (H&E) staining indicated increased number of keratinocytes 3 or 5 days after activin B treatment (Fig. 1C). Activin B administration promoted re-epithelization and hair follicle regeneration after 6 days. SEM analysis further confirmed newly formed epithelial tissue and epithelial cells distributed along collagen on the surface of the healed wound area after activin B treatment (Fig. 1D). Numerous epithelial cells were found on the wound area cross-sections of activin B-treated samples. Furthermore, reduced cell-cell contacts and increased intercellular spaces were observed 5 days after activin B treatment, as revealed by TEM analysis (Fig. 1E). Together with our previous studies demonstrating that activin B could induce migration of mouse primary keratinocytes *in vitro* [9], these data suggest that activin B has the ability to stimulate migration of basal cells both *in vitro* and *in vivo*. Hair follicles also emerged in the regenerated skin around the wound 5 days after activin B treatment.

### Activin B stimulates the proliferation of keratinocytes and hair follicle cells

**Figure 2:**
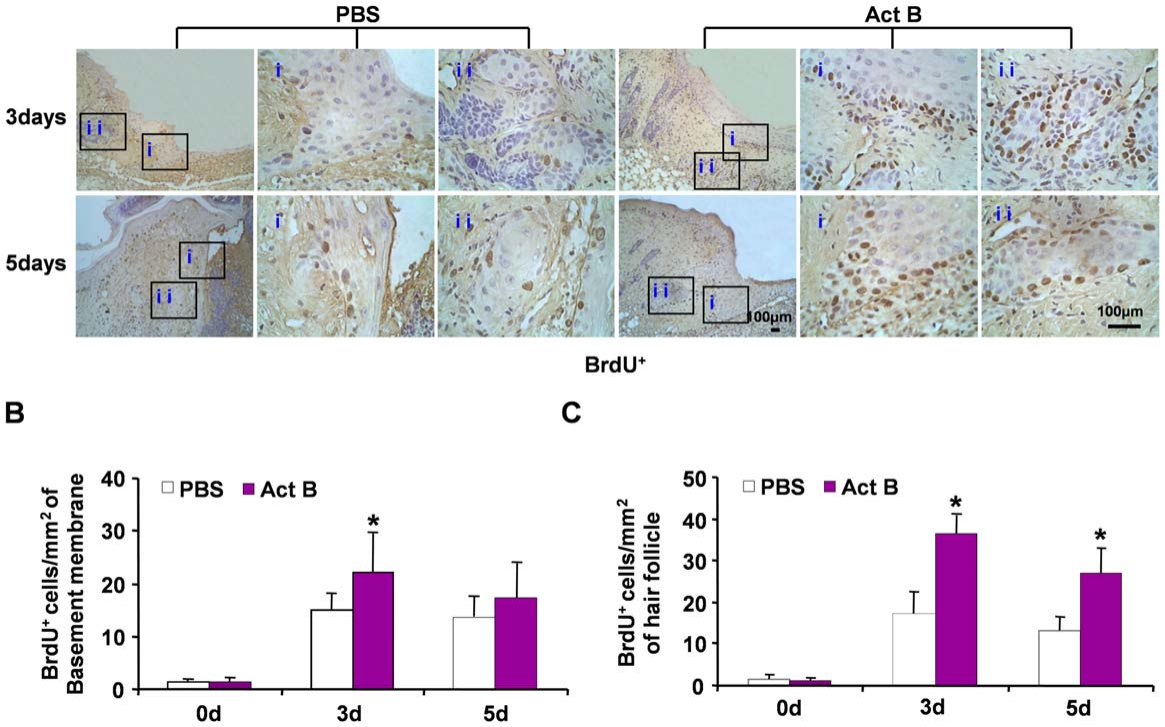
Activin B enhanced the proliferation of keratinocytes and hair follicle cells in wounded skin. Twelve mice were randomly assigned to PBS group and Act B group (n = 6). (A) Immunohistochemistry was performed on the indicated day after PBS or activin B treatment. The BrdU-positive cells were visualized on both epithelium (i) and hair follicle (ii), pictures were taken at 100× magnification, corresponding magnification pictures i and ii were taken at 400× magnification, Scale bars: 100 µm. Data were quantified in three independent experiments and the number of BrdU-positive cells per mm^2^ in epithelium (B) and hair follicle (C) are presented. *P<0.05 compared with PBS control.

Next, we evaluated cell proliferation in wounded skin by BrdU incorporation. The number of BrdU-positive cells increased gradually and was comparable to control and activin B-treated groups during first two days following wound introduction. However, more BrdU-positive cells were observed in both epithelium and hair follicles in activin B-treated mice on day 3 and 5 (Fig. 2), suggesting that activin B enhances the proliferation of epithelial keratinocytes and hair follicle cells during the early stages of wound healing.

### Activin B-induced wound healing is regulated by RhoA

**Figure 3:**
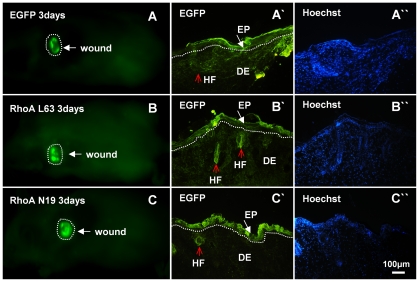
Lentiviral infection of the wounded skin. Six mice were randomly assigned to EGF group, RhoAL63 group and RhoAN19 group (n = 2). After wound creation, 0.2 ml empty vectors (Plenti6/v5-EGFP), RhoAL63 and RhoAN19 with a titer of 1×10^6^ IU/ml were applied to the wound surface. The green fluorescence was visualized by *in vivo* fluorescence imaging system three days after infection (A, B, C), dotted line mean wound. (A′, B′, C′) The green fluorescence on fixed tissue sections was also observed under fluorescent microscope. HF, hair follicle; EP, epithelium; DE, dermis. White arrow indicates epithelium and red arrow indicates the hair follicle. (A″, B″, C″) Sections were double stained with Hoechst 33258 to visualize the nucleus. Scale bars: 100 µm.

**Figure 4:**
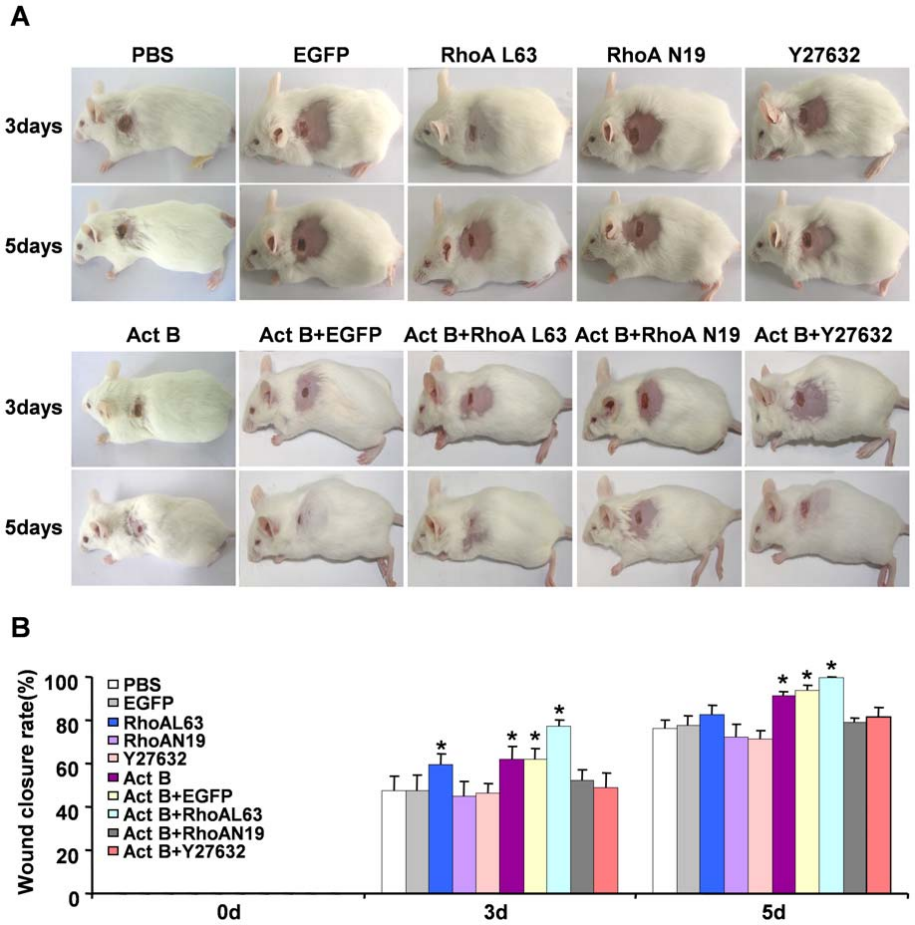
RhoA signal positively regulates the wound closure induced by activin B. Sixty five mice were randomly assigned to eight groups: empty lentiviral vector (EGFP, n = 10), constitutively active RhoA L63 (n = 5), dominant negative RhoA N19 (n = 5), Y27632 (n = 5), Act B+EGFP (n = 10), Act B+RhoAL63 (n = 10), Act B+RhoAN19 (n = 10) and Act B+27632 (n = 10). (A) As described in Materials and methods, animals were treated with Act B+EGFP, Act B+RhoAL63, Act B+RhoAN19, Act B+Y27632 or alone empty lentiviral (EGFP), RhoAL63, RhoAN19 and Y27632 after wound creation. Wound closure status in each group is presented. (B) Wound closure rate calculated on day 0, 3 and 5 after treatment. *P<0.05 compared with PBS control.

**Figure 5:**
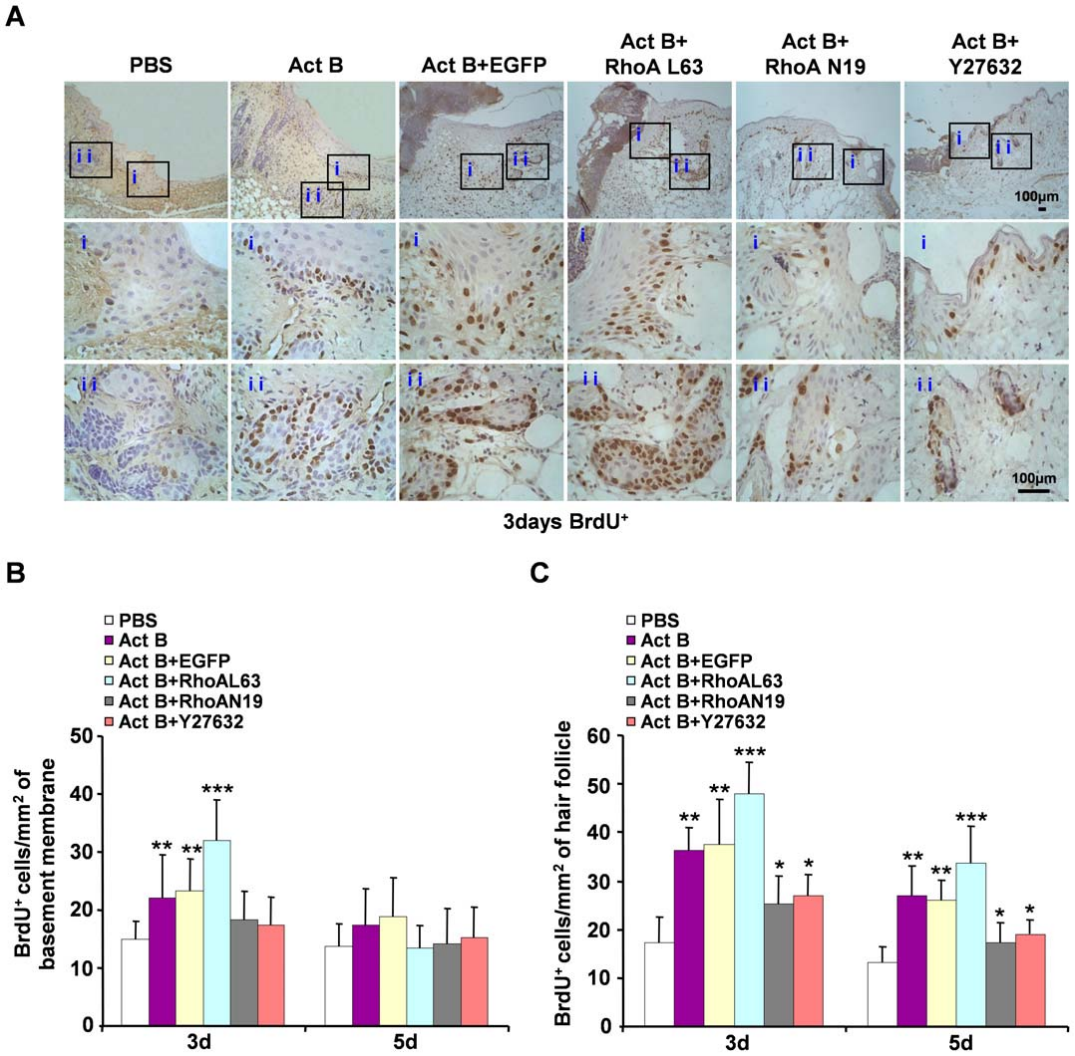
Activation of RhoA contributes to the proliferation of epithelium keratinocytes and hair follicle cells during wound healing. Twenty four mice were randomly assigned to four groups: Act B+EGFP (n = 6), Act B+RhoAL63 (n = 6), Act B+RhoAN19 (n = 6) and Act B+Y27632 (n = 6). (A) Mice were treated with Act B+EGFP, Act B+RhoAL63, Act B+RhoAN19 or Act B+Y27632 after wound creation. Immunohistochemistry was performed on the indicated day after treatment. BrdU-positive cells on epithelium (i) and hair follicle (ii), pictures were taken at 100× magnification, and corresponding magnification pictures i and ii were taken at 400× magnification, Scale bars: 100 µm. Data were quantified in three independent experiments and the number of BrdU-positive cells per mm^2^ in epithelium (B) and hair follicle (C) is presented. *P<0.05 compared with PBS control. **P<0.05 compared with PBS, Act B+RhoAN19 and Act B+Y27632 groups. ***P<0.05 compared with PBS, Act B, Act B+EGFP, Act B+RhoAN19 and Act B+Y27632 groups.

The small GTPase RhoA is essential in activin B-stimulated *in vitro* keratinocyte migration [9]. To investigate the involvement of RhoA signaling in activin B-induced *in vivo* wound healing, wounded skin was infected with EGFP-tagged lentiviruses carrying dominant negative RhoA (N19), constitutively active RhoA (L63) or an empty (control) lentivirus. Three days after empty lentivirus, RhoA N19 and RhoA L63 infection, green fluorescence was detected in epithelial and dermal tissues by *in vivo* fluorescence imaging (Fig. 3A, B, C). Fluorescent images obtained from fixed tissue sections confirmed high viral infection efficiency in skin tissues around the wound site, including the epidermal cell and hair follicle (Fig. 3A′, B′, C′). Interestingly, lentiviral infection with constitutively active RhoA (L63) remarkably enhanced wound healing induced by activin B, while the dominant negative RhoA (N19) infection delayed wound closure. The wound closure rate in the control group was (47.3%±6.5) and (76.1%±4.0) on day 3 and day 5, respectively, whereas wound closure rate was (77.2%±2.8) on day 3 and (99.3%±0.7) on day 5 in RhoAL63-infected plus activin B group, (59.4%±4.9) on day 3 and (82.7%±4.3) on day 5 in RhoAL63-infected alone group, (52.0%±5.0) on day 3 and (78.8%±2.2) on day 5 in RhoAN19-infected plus activin B group, and (44.9%±6.7) on day 3 and (72.1%±5.9) on day 5 in RhoAN19-infected alone group. Furthermore, we assessed the effect of Y27632, a specific inhibitor of the RhoA effector Rock. Wounded skin was treated with 0.2 ml of Y27632 (100 µM) instead of lentivirus prior to activin B administration, or with Y27632 alone. As expected, blockade of RhoA activity by Y27632 inhibited the Act B-accelerated wound healing rate (Fig. 4A, B). In addition, no significant difference was observed in the wound closure rate among empty lentiviral vector infected, PBS-treated skin, RhoN19-infected plus activin B, Y27632 plus activin B, RhoN19-infected and Y27632 alone (Fig. 4A, B). These results demonstrated that RhoA-Rock signaling positively regulated the wound closure induced by activin B. BrdU incorporation experiments further illustrated that compared with activin B group, activation of RhoA by RhoA L63-based lentivirus infection drastically increased the proliferation of epithelium keratinocytes on day 3 (Fig. 5A–C), and hair follicle cells on day 3 and 5 (Fig. 5B, C) during wound healing, whereas infection of dominant negative RhoA (N19) reduced epithelium keratinocytes and hair follicle cells proliferation both on day 3 and day 5 induced by activin B (Fig. 5). However there was no significant difference in epithelium keratinocytes proliferation on day 3 and day 5 in PBS, RhoAN19 plus activin B and Y27632 plus activin B (Fig. 5A, B) groups. In addition, no significant difference was observed in the number of BrdU-positive cells between activin B group and empty lentiviral vector infected plus activin B and between Y27632 plus activin B and RhoAN19 plus activin B (Fig. 5A–C). This suggests that RhoA signaling activation contributes to the epithelium keratinocytes and hair follicle cell proliferation, and therefore stimulated would healing induced by activin B.

### Activin B induces activation of JNK and cJun in wounded skin

**Figure 6:**
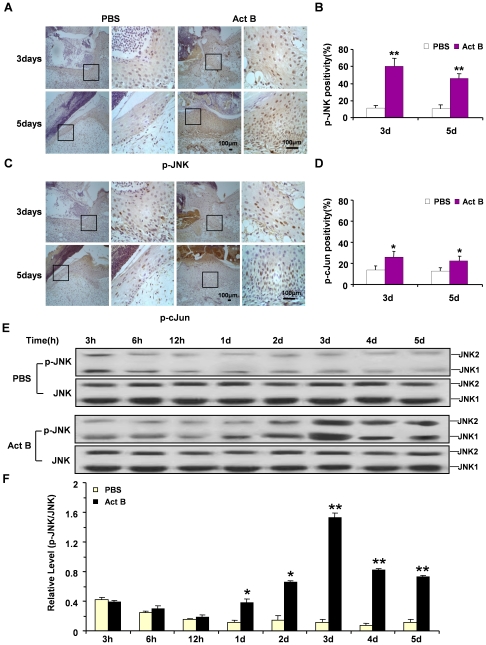
Activin B induced activation of JNK and cJun in wounded skin. Twelve mice were randomly assigned to PBS group and Act B group (n = 6). Immunohistochemistry indicated increased numbers of p-JNK-positive (A) and p-cJun-positive cells (C) at the wounded skin on day 3 and day 5 after activin B treatment, as compared with PBS control. Enlarged images of the boxed area were presented on the right side. Scale bars: 100 µm. Quantified percentages of p-JNK and p-cJun immunopositive cells are presented in (B) and (D) respectively. (E, F) Western blotting showed the level of JNK phosphorylation reached the highest value on day 3 and higher than PBS group on day 1 to day 5. 3 h, 6 h, 12 h: 3, 6 and 12 hours after wound. *P<0.05, **P<0.01, compared with PBS control.

**Figure 7:**
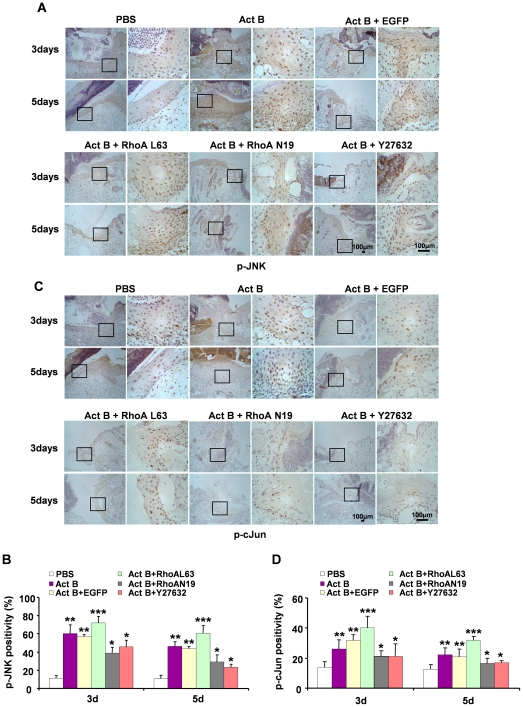
JNK-cJun signaling pathway is involved in activin B-induced wound healing process. Twenty four mice were randomly assigned to four groups: Act B+EGFP (n = 6), Act B+RhoAL63 (n = 6), Act B+RhoAN19 (n = 6) and Act B+Y27632 (n = 6). (A) Mice were treated with empty lentivirus vector (Act B+EGFP), constitutively active RhoA (L63) (Act B+RhoAL63), dominant negative RhoA (N19) (Act B+RhoAN19) or Act B+Y27632 after wound creation. Immunohistochemical staining for p-JNK (A) or p-cJun (C) was performed on indicated day after treatment. Pictures were taken at 100× magnification, and corresponding magnification pictures were taken at 400× magnification, Scale bars: 100 µm. The percentage of p-JNK-positive (B) or p-cJun-positive (D) cells was quantified. *P<0.05 compared with PBS control. **P<0.05 compared with PBS, Act B+RhoAN19, and Act B+Y27632 groups. ***P<0.05 compared with PBS, Act B, Act B+EGFP, Act B+RhoAN19, and Act B+Y27632 groups.

Three major mitogen-activated protein kinases (MAPKs), c-Jun N-terminal kinase (JNK), extracellular signal-regulated kinase (ERK) and p38, control a vast array of physiological processes [12]. Among these MAPK family members, JNK is believed to be a downstream mediator of RhoA-initiated signal transduction pathway. Activation of JNK is required for activin B-induced actin stress fiber formation, *in vitro* keratinocyte migration and wound healing [9]. Compared with the PBS control, increased numbers of p-JNK- and p-cJun-positive cells were found on the wounded skin on day 3 and day 5 after activin B treatment, as revealed by immunohistochemical analysis (Fig. 6A–D). Quantified immunoblot data revealed that after introduction of wound, the phosphorylation of JNK in PBS group was decreased gradually. The levels of JNK activation in Act B group were decreased initially; however, they were increased rapidly from day 1, reaching the highest level on day 3 followed by a gradual decrease. (Fig. 6E, F). Nevertheless, the number of p-ERK- or p-p38-positive cells was not altered (data not shown). Moreover, RhoA activation enhanced JNK activity in the Activin B plus RhoAL63-treated group, while inactivation of RhoA by dominant negative RhoAN19 decreased the activity of JNK (Fig. 7A, B). The Rock inhibitor Y27632 had similar effects as RhoAN19 in suppressing JNK activation (Fig. 7A, B). Similar results were observed in p-cJun activation after different treatments (Fig. 7C, D). These results together indicate that the JNK-cJun signal pathway acts downstream of RhoA-Rock in activin B-induced wound healing process.

### JNK inhibition suppresses activin B-induced wound closure

**Figure 8:**
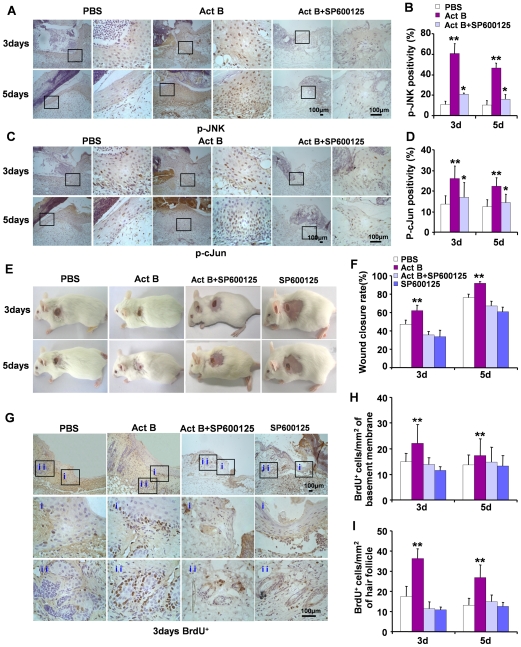
JNK inhibition suppresses activin B-induced cell proliferation and wound closure. Twenty seven mice were randomly assigned to two groups: Act B+SP600125 (n = 16) and SP600125 (n = 11). For JNK inhibition, 0.2 ml SP600125 (5 µM) applied to the wound surface 30 min prior to 0.2 ml of activin B (10 ng/ml) administration, or SP600125 alone. Mice received SP600125 and activin B treatment three times a day. Immunohistochemical staining for p-JNK and p-cJun showed that JNK inhibitor suppressed the expressions of phosphorylated (activated) JNK (A, B) and cJun (C, D) after activin B administration. (E) SP600125 suppressed wound closure induced by activin B and delayed wound healing. (F) Wound closure rate was calculated 3 and 5 days after treatment. (G) BrdU-positive cells were visualized on both epithelium (i) and hair follicle (ii). Pictures were taken at 100× magnification, and corresponding magnification pictures were taken at 400× magnification. Scale bars: 100 µm. Data were quantified in three independent experiments and the number of BrdU-positive cells per mm^2^ in epithelium (H) and hair follicle (I) was presented. *P<0.05 compared with PBS control. **P<0.05 compared with PBS, Act B+SP600125 and/or SP600125 groups.

Since activation of JNK has been observed during wound healing induced by activin B, we asked whether or not JNK activation is required for wound closure. SP600125, which is a specific inhibitor for JNK, was used for this purpose. As revealed by immunohistochemisty staining, SP600125 significantly reduced the level of p-JNK and p-cJun induced by activin B at wound site (Fig. 8A–D), confirming that SP600125 efficiently blocks the JNK-cJun signaling pathway. Importantly, JNK inhibition remarkably suppressed the activin B induced-wound healing process (Fig. 8E, F). The numbers of BrdU-positive cells in both epithelium and hair follicle were dramatically decreased on day 3 and 5 after SP600125 plus activin B and SP600125 alone treatment, as compared with activin B administration alone (Fig. 8G–I). Thus activation of the JNK-cJun pathway is necessary for activin B-induced wound healing by promoting epithelium keratinocytes and hair follicle cell proliferation.

### RhoA mediates wound closure and cell proliferation through JNK signaling after activin B treatment

**Figure 9:**
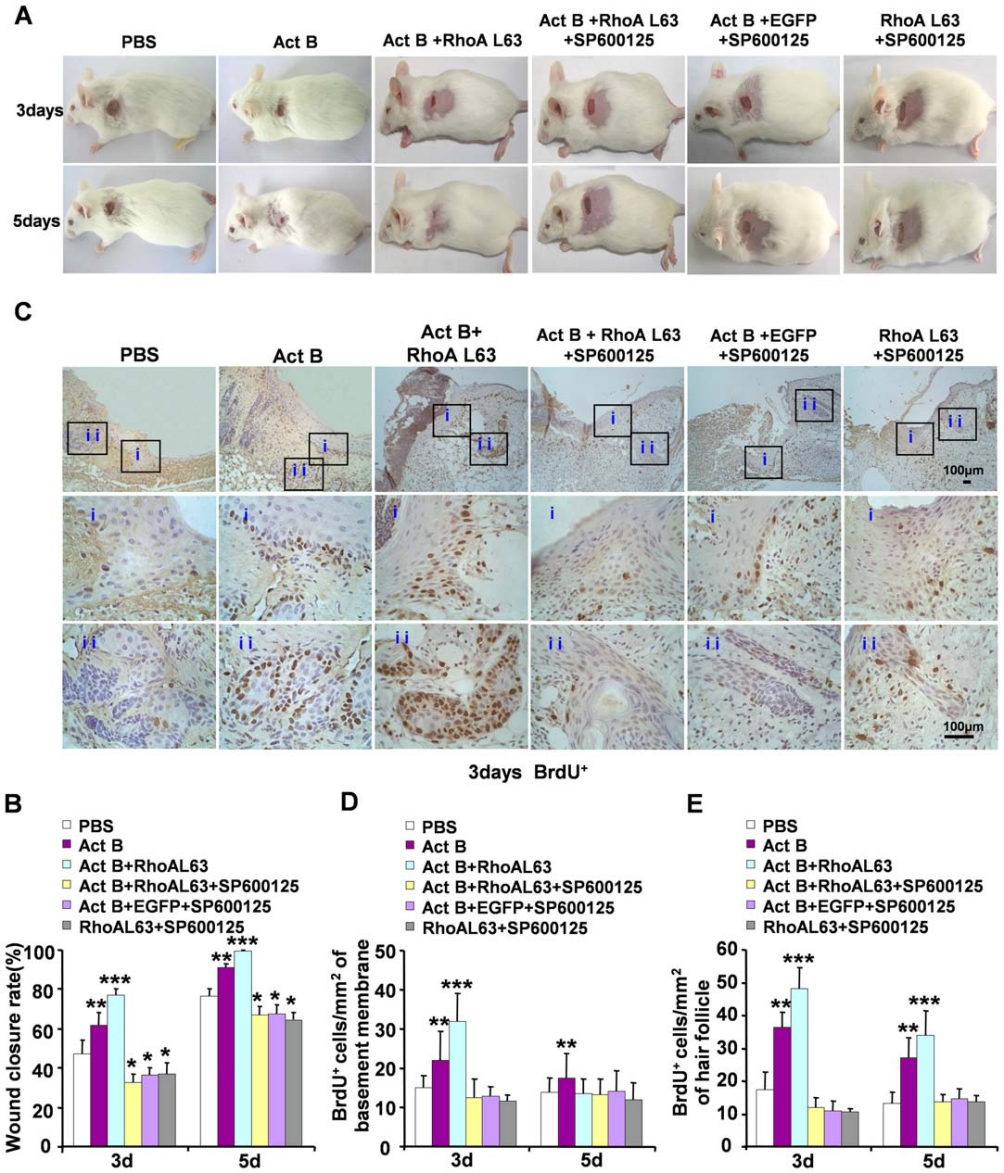
JNK inhibitor efficiently suppresses RhoA activation-promoted wound closure after activin B treatment. Forty three mice were randomly assigned to three groups: Act B+EFGP+SP600125 (n = 16), Act B+RhoAL63+SP600125 (n = 16) and RhoAL63+SP600125 (n = 11). Mice were infected with GFP or RhoAL63 after wound creation. Thirty minutes after lentivirus infection, 0.2 ml SP600125 (5 µM) was injected on the same area. After another 30 min, 0.2 ml of activin B (10 ng/ml) was administered. Mice were received such SP600125 and activin B treatment three times a day. (A) SP600125 suppressed wound closure in mice treated with activin B. (B) Wound closure rate was calculated 3 and 5 days after treatment. (C) BrdU-positive cells were visualized on both epithelium (i) and hair follicle (ii). Pictures were taken at 100× magnification, and corresponding magnification pictures were taken at 400× magnification. Scale bars: 100 µm. Data were quantified in three independent experiments and the number of BrdU-positive cells per mm^2^ in epithelium (D) and hair follicle (E) was presented. *P<0.05 compared with PBS control. **P<0.05 compared with PBS, Act B+RhoAL63+SP600125, RhoAL63+SP600125 and Act B+EGFP+SP600125 groups, ***P<0.05 compared with PBS, Act B, Act B+RhoAL63+SP600125, RhoAL63+SP600125 and Act B+EGFP+SP600125 groups.

To definitively determine the role of JNK in RhoA-mediated wound healing, we investigated the effect of JNK inhibition by SP600125 on RhoA activation (using RhoAL63 lentivirus infection)-promoted wound healing after activin B treatment. On day 3 and day 5 after activin B, RhoAL63 and SP600125 combination treatment, JNK inhibition suppressed wound healing promoted by RhoAL63 and activin B (Fig. 9A, B), and reduced numbers of BrdU-positive cells detected in epithelium and hair follicles (Fig. 9C–E). These observations suggest that JNK is required for RhoA activation-promoted wound closure and cell proliferation after activin B treatment.

### Follistatin suppresses activin B-induced wound healing

**Figure 10:**
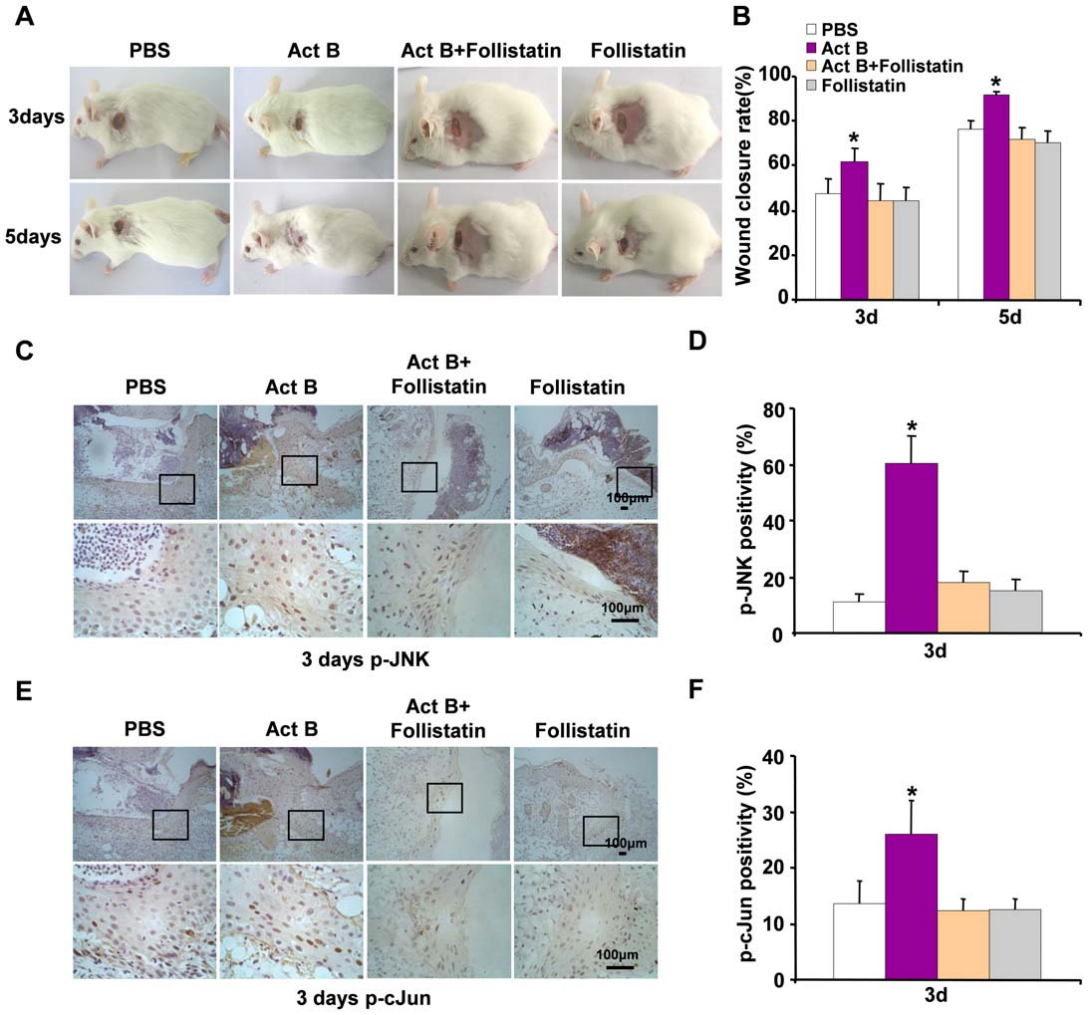
Follistatin efficiently suppresses RhoA activation-promoted wound closure after activin B treatment. Twenty six mice were randomly assigned to two groups: Act B+Follistatin (n = 13) and Follistatin (n = 13). Mice were administered follistatin (0.2 ml 10 ng/ml), thirty minutes after activin B (0.2 ml 10 ng/ml) was administrated, or not. (A) Follistatin suppressed wound closure in mice treated with activin B. (B) Wound closure rate was calculated 3 and 5 days after treatment. Compared with Act B group, follistatin remarkably suppressed the Act B-induced wound healing process. Immunohistochemical staining for p-JNK and p-cJun showed that follistatin suppressed the expression of phosphorylated (activated) JNK (C, D) and cJun (E, F), compared with Act B group. Pictures were taken at 100× magnification, and corresponding magnification pictures were taken at 400× magnification. Scale bars: 100 µm. *P<0.05 compared with PBS control.

The biological activities of activins are regulated, usually inhibited, by follistatin. Follistatin, an activin antagonist, regulates activin activity by binding to activin and inhibiting its binding to receptor [13], [14]. Thus we asked whether follistatin could delay the wound healing rate induced by activin B, and whether follistatin could regulate the activity of downstream signaling JNK-cJun. Wounded skin was treated with 0.2 ml of follistatin (10 ng/ml) prior to activin B administration or with follistatin alone. As expected, follistatin delayed the activin B-induced wound-healing rate (Fig. 10A, B). Compared with the activin B group, decreased numbers of p-JNK- and p-cJun-positive cells were found on the wounded skin on day 3 after activin B and follistatin treatment, or follistatin alone treatment, as revealed by immunohistochemical analysis (Fig. 10C–F). These data together indicate that the activation of the RhoA-JNK-cJun pathway is necessary for activin B-induced wound healing.

## Discussion

Wound repair is a complex process that can be roughly divided into three continuous and overlapping phases: inflammatory reaction, new tissue formation and tissue remodeling [1]. The proliferation and migration of epithelial cells are key events for the formation of epithelium [15], [16]. During this process, keratinocytes migrate to cover the wound surface and fill the wound space, thereby contributing to wound re-epithelialization and healing [2]. Activin B, which is the homodimer subunit of activin, has been recognized to stimulate the wound healing process [7], [8]. However, the molecular mechanism by which activin B mediates wound closure is unclear.

In the present study, we investigated the underlying mechanism by which activin B regulates the *in vivo* wound healing process. Our results demonstrate that activin B increases the number of epithelial cells at the wound edge. During migration, the cell expands by making protrusions, such as large lamellipodia and small filopodia, and contacts with the extracellular matrix (ECM). The rear of the cell must detach from the substratum. Then, the cell exerts a pulling force to translocate the cell body forward and also retracts its rear by regulating actin and myosin [17], [18]. As revealed by TEM analysis, activin B significantly reduced cell-cell contacts and increased intercellular spaces, and the cell rear was detached from the substratum, suggesting that activin B has the ability to stimulate cell migration.

The regeneration of hair follicles during wound repair shares a common process with the hair follicle initiation during the embryonic period [19], [20]. Accumulating evidence has shown that the wound closure rate is closely correlated with scarring response as well as hair follicle regeneration [21], [22], [23]. Most importantly, the regeneration of hair follicles speeds wound closure and reduces scar formation [24], [25], [26]. Here, newly formed hair buds were observed on the basal epithelial tissues and an elevated number of hair follicles were detected three days after activin B treatment compared with the control group. The enhanced regeneration of hair follicles was visible even after 42 days of activin B administration (data not shown). Therefore, it can be concluded that activin B stimulates wound healing by promoting cell migration and hair follicle regeneration. Rho-family GTPases, including RhoA, Cdc42 and Rac1, have recently emerged as important regulators of cell polarity, protrusion and adhesion during directional migration [18], [27]. Activation of matrix metalloproteinase-9 (MMP-9) occurres in response to keratinocyte injury, which is dependent on two distinct pathways: (1) Rac1 and/or Cdc42-mediated activation of p38; (2) RhoA regulated stimulation of JNK [28]. RhoA is required for efficient wound closure of airway epithelial cells [29]. Constitutive activation of RhoA has been found to regulate cytoskeletal reorganization during intestinal epithelial cell spreading [30]. In the present study, wounded skin tissues were successfully infected with RhoA-based lentiviral vetors. Lentivirus infection with the constitutively active RhoA (L63) sequence dramatically promoted the cell migration towards the wound site and accelerated the wound healing process induced by activin B, whereas the dominant negative RhoA (N19) based infection delayed wound closure. These results are consistent with previous reports showing that RhoA contributes to activin-stimulated *in vitro* keratinocyte migration [9]. Collectively, RhoA plays a critical role in mediating activin signals and regulating both *in vivo* and *in vitro* keratinocyte migration.

Emerging evidence suggests that JNK regulates cell migration and its activation is important for wound repair [31], [32], [33]. Gazel et al. reported the activation of the JNK signaling pathway inhibited the differentiation of epidermal keratinocytes and thus stimulated wound healing [34]. Our previous studies indicated that the phosphorylation of JNK induces the migration of keratinocytes and wound repair *in vitro* [11]. Furthermore, activin B-stimulated keratinocyte migration *in vitro* is tightly regulated by a RhoA-MEKK1-JNK signaling pathway [9], [10]. We found that enhanced activation of JNK and cJun occurred during activin B-stimulated *in vivo* wound closure. Blockade of JNK signaling by JNK-specific inhibitor greatly suppressed cell proliferation at the wound site and subsequently delayed wound closure. In addition, activation of RhoA by constitutively active RhoA (L63) based lentiviral infection significantly increased the phorsphorylation of JNK and cJun. In contrast, dominant negative RhoA (N19) based lentiviral infection suppressed the expression of phorsphorylated JNK and cJun. Furthermore, JNK inhibitor was sufficient to delay activin B-induced and RhoA-mediated wound closure. Similarly, the activin antagonist-follistatin remarkably blocked activin B-induced wound healing.

In summary, we demonstrated that activin B promotes *in vivo* epithelial wound healing through a RhoA-Rock-JNK-cJun signaling pathway, which provides important rationale for wound repair therapy. These results also offer some new approaches for solving the problems of hair follicle regeneration during the wound healing process.

## Materials and Methods

### Reagents

Activin B and Follistatin were purchased from R & D Systems (Minneapolis, MN). Primary antibody for rabbit polyclonal p-JNK was obtained from Promega (Madison, WI). Primary antibodies for rabbit polyclonal p-cJun, p-ERK and p-p38 were purchased from Cell Signaling Technology (Boston, MA). BrdU was purchased from Sigma (St Louis, MO), and anti-BrdU antibody was bought from Santa Cruz (Santa Cruz, CA). Goat anti-mouse IgG/Biotin, goat anti-rabbit IgG/Biotin, avidin-biotin-peroxidase complex and diaminobenzidine (DAB) were purchased from Wuhan Boster Biological Technology, Ltd. (Wuhan, China). Specific inhibitors for JNK (SP600125) and RhoA (Y27632) were also obtained from Santa Cruz.

### In vivo wound creation

A total of 247 male and female specific pathogen-free (SPF) level Kunming (KM) mice with body mass of 20 to 25 g, were provided by the Southern Medical University laboratory animal center (SCXK 2006-0015). All experimental procedures were in compliance with the National Institutes of Health guidelines for Care and Use of Laboratory Animals and were approved by the Bioethics Committee of Southern Medical University. For *in vivo* wound creation, 10–12 weeks old mice were anesthetized with an intraperitoneal injection of 10% chloral hydrate (w/v; 0.003 ml/g body mass). Full-thickness skin wounds of 0.5×0.5 cm^2^ were created on each mouse on the dorsal-right or -left shaved skin with sterile scissors as described previously [10]. Wounds were left uncovered after injury, and wound areas were measured at various time points as indicated. The rate of wound closure was calculated using the following formula: Wound closure rate (%) = [(Original wound area-Open area on final day)/Original wound area]×100.

### Lentivirus infection

The lentiviruses bearing dominant-negative mutants RhoA (RhoAN19) and gain of function mutants RhoA (RhoAL63) were generated using general virology techniques, and were named Lenti-RhoAN19 and Lenti-RhoAL63, respectively. All viral preparations were tittered to contain 1×10^6^ infectious units/µl (data not shown). After wound creation, 0.2 ml of viral vector with a titer of 1×10^6^ IU/ml was applied to the wound surface. The infection efficiency was determined using an *in vivo* fluorescence imaging system (lumazone 1024, Roper Scientific, USA) three days after lentivirus infection. The green fluorescence of tissue sections was observed using a fluorescent microscope (IX70, Olympus, Japan).

### Histological examination and immunohistochemistry

Animals were anesthetized and the complete wound with 0.5 cm margins were carefully removed and fixed in 4% paraformaldehyde (PFA), dehydrated with a graded ethanol series, cleared in dimethylbenzene and embedded in paraffin. Five µm sections were deparaffinized by immersing in dimethylbenzene and then rehydrated. H&E staining was performed according to standard procedures. Sections were then subjected to immunohistochemical analysis. Briefly, sections were heated in citrate buffer (0.01 M, pH 6.0) for 5 min at 100°C and were then treated with endogenous peroxidase (3% hydrogen peroxide solution) for 5 min at room temperature. After blocking in 10% goat serum for another 30 min at room temperature, sections were immunostained with primary antibodies for p-JNK (1∶500), p-ERK (1∶250), p-p38 (1∶800), p-cJun (1∶100) or BrdU (1∶50) diluted in phosphate buffer solution (PBS) containing 0.1% Tween-20 and 5% bovine serum albumin (BSA) overnight at 4°C. After washing three times with PBST (PBS supplemented with 0.1% Tween-20), sections were incubated with secondary antibodies, avidin-biotin-peroxidase complex and DAB reagent. Subsequently, all sections were double stained with hematoxylin and visualized under the microscope (BX51, Olympus, Japan). Images were captured by Image-pro plus software, and five-ten photomicrographs were randomly selected from each section. The immuno-positive cells were estimated and presented as a percentage of the total number of cells counted per area. All evaluations were carried out on coded sections. The staining intensity was evaluated in the hair follicle and in the different layers of epidermis [(stratum granulosum, stratum spinosum (SS) and stratum basale (SB)] by using Imageproplus (IPP). The area of interest (AOI) was selected in the hair follicle and epidermis, where was yellow part and showed protein expression. Then the different color in all images was separated by Histogram Based Tool. During color separation, the range of HIS (H means Tone, I means Color Saturation, S means Intensity) respectively was H: 0–40 (p-JNK, p-cJun), H: 0–50 (Brdu**^+^**); S: 0–255; I: 0–255. Finally, we counted the number of protein-positive cells and total cell number by blue hematoxylin staining. The protein positivity (p-JNK and p-cJun) = protein-positive cell number/total cell number×100%. The BrdU-positive cells per mm^2^ = BrdU-positive cell number/the hair follicle or epidermis area.

### Electron microscopy analysis

Animals were anesthetized with intraperitoneal injection of 10% chloral hydrate. The complete wounds with 0.5 cm margins were carefully removed, and washed three times with PBS at 4°C. For scanning electron microscopy (SEM), samples were then fixed with 2.5% glutaraldehyde for 2 days. After dehydration in an acetone gradient [50% (v/v) acetone (10 min), 70% (v/v) acetone (15 min), 90% (v/v) acetone (15 min), 100% acetone (4×15 min)], samples were incubated with isoamyl acetate for 1 h. Spray dried samples were analyzed using SEM (S-3000-N, Hitachi Science Systems, Ltd, Japan). For preparation of transmission electron microscopy (TEM) samples, animals were perfused with 4% PFA and 0.4% glutaraldehyde under anesthesia. Tissues and full-thickness skin from the wound sites (1 mm×1 mm×1 mm) were removed and were post-fixed with osmic acid for 1 h. After dehydration in an acetone gradient, samples were impregnated with a mixture of acetone/resin (1∶1, 1 h, room temperature; 1∶2, 2 h, room temperature) and incubated with resin at room temperature overnight. Sections were stained and images were obtained under an H-7500 TEM (Hitachi Science Systems, Ltd, Japan).

### Western blot analysis

After wound, protein samples were extracted by following the procedures essentially the same as described in detail elsewhere [35]. The protein content was determined with BCA Protein Assay Kit (Beyotime, Haimen, China) using bovine serum albumin as standard. Protein sample (50 µg) was fractionated by SDS-PAGE (10% polyacrylamide gels) and transferred to PVDF membrane (Millipore, Bedford, MA). After blocking in 5% skim milk powder for 2 hours at room temperature, the sample was incubated overnight at 4°C with the Rabbit polyclonal anti-mouse primary antibodies for p-JNK (Promega, Madison, WI), diluted 1∶5000 in TBS containing 5% BSA. After washing three times with TBST (TBS supplemented with 0.1% Tween-20), the sample was incubated 1 hour at room temperature with anti-rabbit HRP-linked second antibodies. Bound antibodies were detected using the chemiluminescent substrate ECL (Millipore, Bedford, MA). Then washing with stripping buffer (Pierce protein, Thermo, Rockford, IL) for 10 min at room temperature and blocking, the sample was incubated overnight at 4°C with the Rabbit polyclonal anti-mouse primary antibodies for JNK (Cell Signaling Technology, Boston, MA), diluted 1∶1000 in TBS containing 5% BSA, and was incubated with second antibodies and detected. Western blot bands were quantified using QuantityOne software by measuring the band intensity (Area×OD) for each group and normalizing to JNK as an internal control. The final results are expressed as fold changes by normalizing the data to the control values.

### Statistical analysis

Data were analyzed using SPSS 13.0 software and plotted as mean ± SD. Wound closure rate was evaluated by single factor repeated measurement. Statistical significant differences were carried out by one-way ANOVA.
